# Duration of Lactation and Maternal Risk of Metabolic Syndrome: A Systematic Review and Meta-Analysis

**DOI:** 10.3390/nu12092718

**Published:** 2020-09-05

**Authors:** Christine Tørris, Ann Kristin Bjørnnes

**Affiliations:** Health Sciences, Oslo Metropolitan University, 0130 Oslo, Norway; anki@oslomet.no

**Keywords:** breastfeeding, lactation, maternal health, women, gender, metabolic syndrome, insulin resistance, cardiovascular risk, cardiovascular disease

## Abstract

Cardiovascular disease (CVD) is the leading cause of death of women across all ages, and targeting modifiable risk factors, such as those comprised in metabolic syndrome (MetS) (e.g., waist circumference, lipid profile, blood pressure, and blood glucose), is of great importance. An inverse association between lactation and CVD has been suggested, and lactation may decrease the risk of MetS. This systematic review and meta-analysis examined how lactation may affect the development and prevalence of MetS in women. A literature search was performed using Cinahl, Embase, Web of Science, and PubMed. A total of 1286 citations were identified, and finally, ten studies (two prospective and eight cross-sectional) were included. Seven studies (two prospective and five cross-sectional) revealed associations between lactation and MetS, suggesting that breastfeeding might prevent or improve metabolic health and have a protective role in MetS prevention. This protective role might be related to the duration of lactation; however, a lack of controlling for potential confounders, such as parity, might inflict the results. The pooled effect was non-conclusive. Additional research is required to further explore the duration of lactation and its potential role in improving or reversing MetS and its components.

## 1. Introduction

Globally, cardiovascular disease (CVD) is the leading cause of death of women across all ages [1]. Despite the death rates from heart disease have been decreasing for decades, recent data demonstrate a continued but slower decline in age-adjusted mortality rate after 2010 [2], and recent data show an increase in CVD incidence and deaths among women 45 to 54 years of age [3,4,5]. Although men suffer CVD at an earlier age and a higher age-specific rate than women, women have been shown to have several disadvantages in terms of CVD compared to men. These inequalities are associated with a higher cardiac risk factor burden in women, including a higher prevalence of systemic inflammatory rheumatologic diseases, mental stress/depression, polycystic ovarian syndrome, and factors related to reproductive health (e.g., pregnancy-induced hypertension, preeclampsia, gestational diabetes) [6,7,8].

The underlying disease process of CVD is atherosclerosis, a complex inflammatory process in the walls of blood vessels. Female sex hormones are suggested to drive systemic inflammation; however, other factors related to female sex may reduce the vascular impact of enhanced systemic inflammation in women. Still, sex differences in atherosclerosis are not rigorously addressed in clinical studies [9]. Interestingly, an inverse association between history of lactation and CVD incidence has been suggested [10,11]. Schwarz et al. [10] reported that women without a history of lactation had approximately three times higher odds for aortic calcification (OR 3.85, 95% CI: 1.47–10.00) and coronary artery calcification (OR 2.78, 95% CI: 1.05–7.14) compared to mothers who breastfed for more than 3 months. According to a large US cohort study (*n* = 139,681; median age 63 years) [12], a lifetime history of more than 12 months of lactation was associated with reduced cardiac risk factor burden, including hypertension, diabetes, and hyperlipidemia, and reduced occurrence of CVD (OR 0.91, *p* = 0.008) compared to women without a history of lactation.

In 2016, 40% of women globally, aged >18 years, were overweight [13], and women who enter menopause, being overweight or obese, are at great risk for cardiometabolic disruptions [14]. Metabolic syndrome (MetS) is a cluster of risk factors for CVD, consisting of metabolic abnormalities, such as abdominal obesity, dyslipidemia, hypertension, and hyperglycemia [15,16]. MetS is a major public health problem that increases the risk of both morbidity and mortality [17] and has been associated with a doubling of CVD risk and a five-fold increased risk of diabetes mellitus type 2 (DM2) [18]. The prevalence of MetS is high and has been found to affect 17% to 46% of the general population in the developed world, with an increasing prevalence with age [19]. To reduce the number of CVD events in women, targeting the modifiable cardiovascular risk factors comprised of MetS (e.g., waist circumference, lipid profile, blood pressure, and blood glucose) is of great importance [16].

In women, several factors may impact the prevalence and characteristics of MetS, such as childbearing and menopause [20]. Childbearing is associated with changes in the maternal metabolic system, such as weight gain and increased central adiposity [21], and both parity and an increasing number of children have been associated with a higher risk of MetS [22,23,24,25,26]. Lactation, on the other hand, has been found to decrease the prevalence of MetS [22].

Over the past decades, several health benefits from breastfeeding for both women and child have been revealed [27], and today it is recommended that infants should be exclusively breastfed for the first six months of life [28], and thereafter for one or two years in addition to complementary foods. In women, physical changes that occur during pregnancy (e.g., visceral fat accumulates, insulin resistance, increased lipid levels) may reverse more quickly and more completely with lactation [29,30]. Furthermore, prolonged lactation may be associated with a healthier metabolic profile and body composition, especially lipid levels and waist-to-hip ratios [27,31,32]. According to a systematic review by Zachou et al. [33], lactation has a protective effect against the development of hypertension, diabetes, and possibly CVD. Still, the mechanisms underlying the link between lactation and cardiometabolic risk profile are unclear.

Few studies have investigated associations between breastfeeding and MetS development and prevalence in women. Therefore, it is important to elucidate possible preventive effects of lactation on maternal cardiovascular health, especially related to women’s risk of MetS after pregnancy and lactation. This study primarily aimed to examine whether women’s breastfeeding duration may reduce the development and prevalence of MetS in women. This research question was explored by reviewing primary research studies conducted among women (humans) and reporting breastfeeding as being related to MetS (prevalence or incidence), where MetS was defined via an established definition.

## 2. Materials and Methods

To identify published studies examining associations between having breastfed in adult women as the exposure and the development and prevalence of MetS as the outcome, literature searches were performed in Cinahl, Embase, Web of Science, and PubMed. The combined search terms were (1) breastfeeding and metabolic syndrome, (2) lactation and metabolic syndrome utilized as MeSH (Medical Subject Headings) and keywords in accordance with the different database thesaurus. The searches were performed in March 2020.

**Figure 1:**
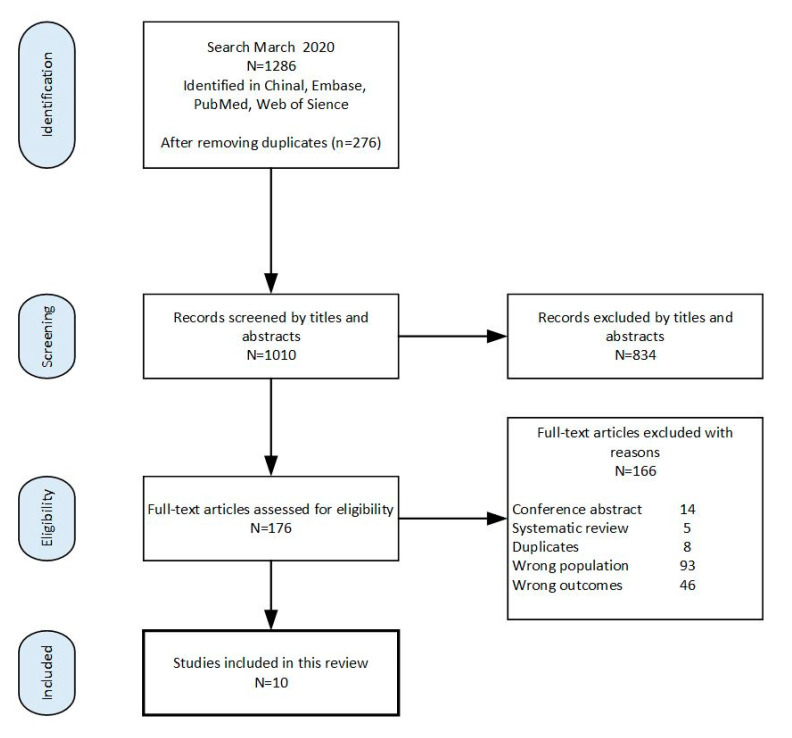
Flow diagram of the review process.

Duplicates were removed, and potential abstracts were independently screened against the eligibility criteria by the two review authors. Thereafter, all relevant abstracts were independently re-assessed for eligibility using full-text reports, and finally, ten studies were included in this systematic review after exclusion. The flow diagram of the review process is shown in Figure 1.

All cross-sectional, prospective cohort and intervention studies conducted among adult women (humans), reporting duration of breastfeeding related to MetS (prevalence or incidence) as an entity using established definitions were considered for inclusion. Abstracts, letters, or reviews were not included but inspected for additional references meeting the inclusion criteria. Additionally, the reference lists of the included studies and relevant published reviews were examined to identify additional papers for possible inclusion. A summary of the selection criteria (participants, interventions, comparators, and outcomes) was considered according to the PICOS (Population, intervention, comparison, outcome, study designe) strategy and is provided in Table 1.

The search was restricted to papers written in English and to studies conducted among humans, and animal studies were excluded. The full text of the article was retrieved whenever there was any uncertainty. Disagreements in the inclusion process were resolved by discussion between the two review authors; if no agreement could be reached, it was planned that a third author would decide. However, this was not necessary. The included studies were assessed according to the quality of their reporting of study design and method(s), as well as statistical analysis. The procedure for the review was carried out in accordance with the PRISMA statement for review reporting [34], and a protocol of the study selection was made. The PRISMA statement is provided in Supplementary Materials File S1.

The data collected from the studies were a reference, the country where the study was performed, design, aim, sex, participants’ age (baseline age and duration of follow-up for prospective studies), sample size, methods of measurement, variables adjusted for in the analysis, multivariate-adjusted odds ratio (OR) with a 95% CI.

### 2.1. Quality Appraisal

The methodological quality (i.e., risk of bias) assessment was performed using the Joanna Briggs Institute (JBI) checklist for cross-sectional studies and cohort studies, as appropriate [35]. The JBI appraisal checklist for cross-sectional studies includes eight items, and the checklist for cohort studies includes 11 items to assess the study‘s internal validity, and both checklists are well established and frequently used globally [36].

### 2.2. Statistical Analysis

When appropriate, dichotomous patient data related to the incidence of MetS at 12 months were pooled to compare women who breastfed with women with no history of breastfeeding to determine the overall effect of breastfeeding on MetS. The results for the dichotomous data were presented using the relative risk difference [37], and the analysis was performed using the Cochrane Collaboration‘s Review Manager Software (RevMan, version 5.3). We were not able to extract continuous data as the included studies lack the appropriate information. Instead, a forest plot was constructed by utilizing the appropriate features in Microsoft Excel software to visualize the adjusted odds ratio for MetS associated with lactation identified in the included studies.

## 3. Results

### 3.1. Literature Search Results

Initially, 1286 citations were identified, and 1010 abstracts were screened after duplicates were removed. After screening titles/abstracts, 176 full-text articles were retrieved and re-assessed for eligibility. The main reasons for exclusion were the studies investigating the association in children, missing data on duration of maternal breastfeeding, or no established definition for MetS. Finally, ten studies met the inclusion criteria and were included in this meta-analysis. The flow diagram of the review process is shown in Figure 1. The included studies comprised of two prospective studies (Table 2) and eight cross-sectional studies (Table 3), of which two prospective studies [38,39] and five cross-sectional studies [22,40,41,42,43] revealed an association between duration of lactation and MetS. 

### 3.2. Prospective Studies

Gunderson et al., 2010 [38], investigated the association between duration of lactation and incidence of MetS in a 20-year prospective study from the U.S. At baseline, 1399 nulliparous women (39% black, aged 18–30 years) were included from the Coronary Artery Risk Development in Young Adults (CARDIA) Study. To examine the association of lactation duration and incidence of the MetS, 704 women who delivered at least one singleton, live birth during the 20-year period (1986–2006) were included. The women reported the number of months of lactation for each pregnancy (i.e., none, <6 weeks, 6–11 weeks, 3–6 months, for >6 months), and the midpoint of each lactation category was assigned (i.e., 21 days for <6 weeks, 66 days for 6–11 weeks, 135 days for 3–6 months, and 210 days for >6 months) (Table 4). The total duration of lactation for each time interval was obtained by summing the number of days of lactation across all births within an interval. Then, the total duration of lactation was classified into one of four lactation groups for each time interval: 0–1 month, >1–5 months, 6–9 months, and >9 months, representing the cumulative lactation duration for all births since baseline. Within the gestational diabetes mellitus (GDM) and non-GDM groups, the referent group was 0–1 month. MetS was identified according to the National Cholesterol Education Program Adult Treatment Panel III (NCEP ATP III) criteria [47]. The incidence of MetS increased over time as the cohort aged (non-GDM 8/382 (2.1) to 46/463 (9.9), GDM 6/43 (14.0) to 9/55 (16.4)), with the greatest increase between years 15 and 20 compared with the earliest interval. The cumulative incidence of MetS was highest for 0–1 month regardless of GDM status. Overall, there was a six-fold lower crude incidence rate (number of case participants per 1000 person-years) of MetS for >9 months versus 0–1 month of lactation among the GDM group (49.4 vs. 8.5) and a two-fold lower incidence rate for the highest versus lowest duration of lactation among the non-GDM group (16.7 vs. 9.2). The duration of lactation was inversely associated with the relative hazards of incident MetS, from >1–5 months to >9 months compared with 0–1 month, with a stronger inverse association among GDM (relative hazard 0.11–0.24) than non-GDM groups (relative hazard 0.41–0.49) in unadjusted models (all *p* < 0.001). The significant inverse association remained for GDM and non-GDM groups, both after adjusting for race, time-dependent parity, study center, and baseline covariates (age, education, and smoking), and in the fully adjusted models, the addition of baseline BMI, all MetS components, and physical activity (GDM: relative hazard 0.14–0.33, non-GDM relative hazard 0.44–0.61) (all *p* = 0.03).

Ramezani Tehrani et al., 2014 [39], investigated associations between duration of lactation and the development of MetS in a 9-year prospective study from Iran, where 925 women without MetS at baseline were randomly selected from the Tehran Lipid and Glucose Study (aged 15–50 years) [39]. Duration of lactation was reported in questionnaires, and the women were assigned to five groups according to their lifetime duration of lactation: none, 1–6 months, 7–12 months, 13–23 months, and 24 months or more. Of the 340 women that reported no lactation, 311 were nulliparous, and 29 parous. In the four lactating groups, the women had, on average, two children. MetS was defined using the clinical diagnostic criterion for Iranian adult MetS [48]. After nine years, the incidence of MetS was 12.1% in the non-lactating group, and 28.6%, 34.0%, 26.2%, and 26.7% in women who breastfed for 1–6, 7–12, 13–23, and 24 months or more, respectively. When comparing the groups, no lactation was associated with the reduced risk of MetS, compared to lactation for 24 months or more, RR 0.3 (95% CI 0.2–0.5), indicating that negative history of lactation was associated with decreased MetS. However, the direction of this association was reversed and not significant after adjusting for age, physical activity, daily caloric intake, BMI, and parity (RR 1.5, (95% CI 0.7–3.0)). Among the four lactating groups, no significant differences in the incidence of MetS were revealed. However, in the adjusted model, a significant association between 13 and 23 months duration of lactation and higher incidence of MetS were found, compared with 24 months or more, RR 1.8 (95% CI 1.0–3.4), indicating that a longer duration of lactation for more than two years was associated with decreased MetS.

### 3.3. Cross-Sectional Studies

Cho et al., 2009 [44], investigated associations between reproductive factors and MetS in 892 postmenopausal women from the Korean National Health and Nutrition Examination Survey (KHANES) 2005 [44]. Lactation was defined as a history of breastfeeding for at least one month, and the participants were assigned to one of two categories regarding lactation: ever and never. MetS was defined using the NCEP ATP III criteria III JAMA 2001, except abdominal obesity, where the criterion was adopted from the Korean Society for the Study of Obesity [49] (Table 5). MetS was present in 31% of the women. The mean (SD) differed and was statistically significant between the women with MetS compared with those without MetS; age 65.2 (9.01) vs. 62.43 (8.87), years since menopause 15.05 (9.99) vs. 12.34 (9.66), parity 4.02 (2.01) vs. 3.60 (1.89), respectively. The study found no association between lactation and MetS, unadjusted OR 1.48 (95% CI 0.84–2.60), and adjusted OR 1.20 (95% CI 0.65–2.20) (age, body mass index, and demographic, socioeconomic, and lifestyle factors).

Choi et al., 2017 [40] investigated the association between duration of breastfeeding and MetS in 4724 parous non-pregnant women (19–50 years) from the Korean National Health and Nutritional Survey (KNHANES) 2010–2013 [40]. Duration of lactation was collected through an open-ended questionnaire, and the women were divided into four groups according to their total lifetime duration of breastfeeding: ≤5, 6–11, 12–23, or ≥24 months. MetS was defined according to NCEP-ATP III [50], except for waist circumference, where the International Obesity Task Force criterion for Asian-Pacific populations (<80 cm) was used (Table 5). The women were, on average, 40 years and had a 13% prevalence of MetS. The study revealed a lower risk of MetS among those breastfeeding for ≥12 months, compared with those breastfed for ≤5 months. Women who breastfed for 12–23 months had a 27% decreased risk of MetS, and those breastfeeding for ≥24 months had a 30% decreased risk of MetS (adjusted for age, BMI, household income, educational level, marriage status, smoking status, alcohol drinking, physical activity, age at menarche, menopause, parity, and use of oral contraceptives). Of the MetS components, especially decreased blood pressure (BP), glucose, and triglyceride were associated with a longer duration of breastfeeding. Women who breastfed for 12–23 months had a 32% decreased risk of elevated BP (95% CI, 0.54–0.86) and a 22% decreased risk of elevated glucose (95% CI 0.62–0.97). Women who breastfed for ≥24 months had a 38% decreased risk of elevated glucose (95% CI 0.52–0.84) and a 24% decreased risk of elevated TG (95% CI 0.60–0.96), compared with women who breastfed for ≤5 months. In addition, women who breastfed for 6–11 months had a decreased risk of having elevated blood pressure (BP), OR 0.67 (95% CI 0.51–0.89), compared with women who breastfed for ≤5 months.

Cohen et al., 2006 [22] investigated the association between reproductive factors (parity and breastfeeding) and MetS in women from the Third National Health and Nutrition Examination Survey (NHANES III) [22]. A total of 4699 women, aged ≥20 years, were included in the study. The women were of different ethnicity (non-Hispanic white, non-Hispanic black, Mexican, American, or other). Parity was reported as the number of live births, and the breastfeeding as ever breastfeeding for at least 1 month. MetS was defined according to ATP III. The study revealed a significant association between duration of lactation and MetS, where women who lactated for ≥1 month had a 22% lower risk of MetS compared to those lactating for less than one month (adjusted for age, race/ethnicity, income, education, sociodemographic, reproductive, and behavioral risk factors). However, the odds of MetS increased by 13% with each additional child.

Ki et al., 2017 [41] investigated the relationship between duration of breastfeeding and obesity in postmenopausal women (*n* = 6621) from the Korea National Health and Nutrition Examination Survey (KNHANES) 2010–2012. The duration of breastfeeding was calculated by dividing the total period of breastfeeding by the number of breastfed children, and then divided into groups (<1 month, 1–6, 7–12, 13–18, and >18 months per child). The study revealed a lower prevalence of MetS in women who breastfed ≥ 6 months compared to those who had not breastfed (29.5 (2.1) vs. 88.4 (4.6), *p* = 0.056).

Kim et al., 2016 [45] investigated the association between breastfeeding and MetS in women from the fifth Korea National Health and Nutrition Examination Survey (KNHANES) 2010. A total of 1053 parous women aged 30–49 years (542 in their 30s and 511 in their 40s) participated in the study. The duration of breastfeeding was reported in months, and the breastfeeding experience as yes or no. MetS was defined using the Joint Interim Statement (JIS), except for abdominal obesity, where the criterion from the Korean Society for the Study of Obesity in 2006 was used. MetS was present in 5.1% of the women in their 30s and 10.1% of the women in their 40s. No associations between breastfeeding duration and MetS were revealed, neither in women in their 30s OR 0.99 (95% CI 0.95–1.02) nor in women in their 40s OR 0.98 (95% CI 0.94–1.02) (adjusted for income, education level, exercise, and the last childbirth age). In women in their 30s, breastfeeding experience (yes/no) was associated with the reduced risk of MetS OR 0.28 (95% CI 0.10–0.77); however, it was not significant in the adjusted model OR 0.37 (95% CI 0.14–1.03). Breastfeeding more children was associated with the increased risk of MetS, unadjusted OR 4.17 (95% CI 2.10–8.26), and adjusted OR 4.03 (95% CI 2.03–8.00).

Moradi et al., 2016 [46] investigated associations between duration of breastfeeding and MetS in Iranian women who had at least one previous live birth (*n* = 978, aged 40–70 years) [46]. The duration of lactation was reported as total lifetime lactation duration (months). MetS was defined according to the ATP III definition [51]. The mean age (SD) of the women was 53 (7.85) years, the mean age at first parity was 19.27 (4.13), the mean number of pregnancies was 4.71 (2.26), and the mean duration of lactation was 81.55 (51.22) months. The study revealed no statistically significant associations between breastfeeding duration and MetS, OR 0.99 (95% CI 0.99–1.00) (adjusted for age, age at first pregnancy, number of pregnancies, and history of DM and hypertension).

Ram et al., 2008 [42] investigated associations between duration of lactation and the prevalence of MetS in women from the Study of Women’s Health Across the Nation (SWAN) [42]. The study included 2516 US women (aged 42–52 years) who reported a live birth, whereof 1620 had a history of breastfeeding. The women were of different ethnicity, and for each Caucasian woman, a non-Caucasian (African American, Hispanic, Chinese, and Japanese) woman was recruited. Participants answered retrospective questions about the number of pregnancies and lactation duration following each live birth. The duration of lactation was coded in months, with less than 1 month of lactation coded as zero, and for women who breastfed longer than one year/pregnancy, each lactation interval was truncated at 1 year. MetS was defined according to the National Cholesterol Education Program (NCEP) III criterion [51]. The study revealed a significant association between duration of lactation and a 20% lower risk of MetS for each year of lactation (unadjusted). The association remained significant after adjusting for age, smoking history, parity, ethnicity, study site, socioeconomic status, physical activity, daily caloric intake, and high school BMI, OR per each additional year of lactation 0.88 (95% CI: 0.77–0.99). In addition, ever having breastfed was associated with a 23% decreased risk of MetS (adjusted OR 0.77 (95% CI 0.62–0.96)), compared to not having breastfed. Of the MetS components, especially elevated blood pressure (OR 0.90 (95% CI 0.81–0.996)) and abdominal obesity (OR 0.86 (95% CI 0.78–0.96)) were inversely associated with the duration of lactation.

Yu et al., 2019 [43] investigated associations between breastfeeding on markers of cardiovascular disease risk in 622 women with pregnancy complications (hypertensive disorder, gestational diabetes, intrauterine growth restriction, abruption, or preterm birth), 6 months postpartum. Self-reported breastfeeding status and the measured cardiovascular (CV) risk factors were assessed. The prevalence of MetS was compared between women who did not breastfeed (*n* = 100, 16%), those who breastfed for less than 6 months (*n* = 315, 51%), and those who breastfed for 6 months or more (*n* = 207, 33%). MetS was defined, according to Kahn et al. 2005 [52]. The study revealed that increased breastfeeding duration decreased the prevalence of MetS (adjusted OR 0.89 (95% CI 0.79–0.99)).

### 3.4. Criteria for Duration of Lactation

Lactation was categorized differently in the included studies, which is presented in Table 4.

### 3.5. The Definition of Metabolic Syndrome

The included studies used different definitions of MetS for waist circumference and blood glucose, where the waist circumference criteria values ranged from 80 to 95 cm (Table 5). For blood glucose, Cohen et al., 2006 [22], Ki et al., 2017 [41], and Ram et al., 2008 [42] used ≥6.1 mmol/L (110 mg/dL), while the rest of the included studies used ≥5.6 mmol/L (100 mg/dL). For S-triglyceride (>1.7 mmol/L (150 mg/dL)), S-HDL cholesterol (<1.3 mmol/L (50 mg/dL)), and blood pressure (≥130/85 mmHg), the criteria values were the same in all the included studies, even if some of the studies included drug treatment as a criterion for these conditions.

### 3.6. Quality Appraisal

Most of the included studies were limited by design as 80% (*n* = 8) of the ten studies included were utilizing a cross-sectional design, and the overall methodological quality was moderate. The risk of bias was primarily related to unclear identification of confounding factors (*n* = 3, 38%), unclear description of inclusion/exclusion criteria (*n* = 4, 50%), unclear description of study participants (*n* = 5, 63%), and unclear description of the outcome measures. For the cohort studies, it was unclear if the groups were similar at baseline (*n* = 1, 50%), unclear identification of confounding factors (*n* = 1, 50%), and unclear if the follow-up was complete (*n* = 1, 50%). The risk of bias table for each of the included studies is provided in Supplementary Materials File S2.

### 3.7. Meta-Analysis

**Figure 2:**
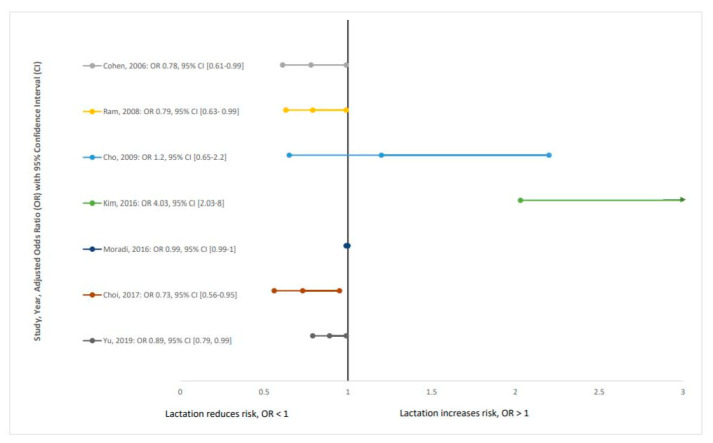
Adjusted odds ration with 95% confidence intervals across studies.

Seven articles presented information about adjusted OR for MetS, and Figure 2 illustrates the association between lactation and MetS. Of these, four studies [22,40,42,43] presented data that indicated a statistically significant lower OR for MetS among women who were breastfeeding.

**Figure 3:**
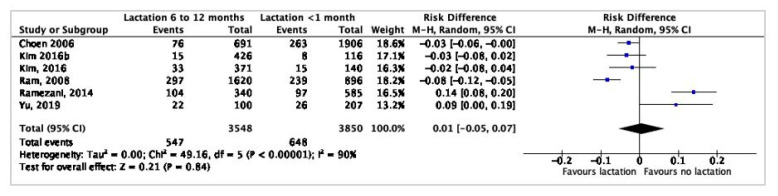
Forest plot.

Figure 3 presents a meta-analysis of the dichotomous data from five studies [22,39,42,43,45] about MetS prevalence among women who breastfed up to one year and MetS prevalence among women who breastfed shorter than one month. The heterogeneity between studies was high and was minimized by using a random effect model. The pooled results were inconclusive as the risk difference between the groups was 0.01 (95% CI 0.05–0.07).

## 4. Discussion

### 4.1. Summary of Main Results

Even when most of the included studies revealed beneficial associations between lactation and MetS, the pooled effect was non-conclusive. Despite data scarcity, the results suggest that lactation may have a preventive role in MetS development as two prospective studies [38,39] and five cross-sectional studies [22,40,41,42,43] revealed an association between duration of lactation and MetS, indicating that lactation improves metabolic health, where especially the longer duration of lactation may have a protective effect.

The included studies used different criteria for waist circumference and blood glucose when diagnosing MetS. To use different thresholds for abdominal obesity in different ethnic populations is recommended; however, it has been questioned if the same criterion should be applied to ethnic groups regardless of their country of residence [16]. In a multi-ethnic sample population, this may be a challenge. Among the included studies, several of them seem to have included a sample population with the same ethnic background; however, used different thresholds. The use of different definitions may lead to a higher or lower prevalence of MetS, which may have influenced the results in the included studies.

The incidence of MetS increased over time in both prospective studies; however, in the study of Ramezani Tehrani et al., 2014 [39], the incidence was lower among those who never breastfed compared to those who had. In women, the risk of MetS and its components increases with each pregnancy [11,12], and likely the women who never breastfed, in the study of Ramezani Tehrani et al., 2014 [39], had a lower MetS incidence as most of them did not have children (mean (SD) 0 (0.3)), compared to approximately those with two children (mean (SD) 1.8 (1.0), 2.1 (1.1), 1.9 (1.1), 2.3 (1.1)) who breastfed for 1–6 months, 7–12 months, 13–23 months, and ≥24 months, respectively. The increased risk of MetS with parity was also revealed by Cohen et al., 2006 [22], who found that the odds of MetS increased by 13% with each additional child, especially in women not of non-Hispanic black race/ethnicity.

Prolonged lactation may be associated with a healthier metabolic profile and body composition, especially lipid levels and waist-to-hip ratios. Previous studies have observed inverse associations between prolonged lactation and the components of MetS—waist circumference, blood pressure, s-triglycerides [53], and DM2 [54,55,56,57].

### 4.2. Potential Mechanisms

In pregnancy, changes in the woman’s metabolism occur as she accommodates to meet both her own and the child’s nutritional needs [58]. Excessive weight gain during pregnancy is common. A US study revealed that as much as 40% of normal-weighted and 60% of over-weighted women gained excessive weight during pregnancy [59]. After birth, lactation increases the mother’s energy requirement, with approximately 2 MJ per day [60]. Interestingly, higher energy intake has been revealed several years after weaning, where longer duration of lactation has been associated with slightly higher energy intake (0.7 MJ per day), with a significantly higher intake of protein and fiber, and a higher level of physical activity, several years after weaning (range: 0.7–13.9 years) [61].

Lactation may help women to regain their prepregnant weight and metabolic and cardiovascular risk status, as the “reset hypothesis” suggests; however, the pre-lactation metabolic risk factors might influence lactation initiation and duration [29]. Women who lactate may return faster to their pre-pregnant weight [62], and weight loss has been observed, especially during the first 12 months after giving birth, with weight retention for 16 months [63]. A greater weight loss has been found with exclusive breastfeeding for six months; however, lactation may also enhance weight loss if the breastfeeding period continues for at least 6 months [63].

Complex and interacting signaling pathways regulate systems of responses within the brain, gut, and adipose tissue and controls the appetite, energy homeostasis, and the maintenance of fat mass. Adipose tissue secretes hormone-like substances, known as adipokines. Adipokines are cytokines, secreted by adipose tissue, which have paracrine and endocrine actions and regulate metabolism, inflammation, and body homeostasis [64]. Through adipokines, the adipose tissue influences the appetite, satiety, energy expenditure, fat storage, and insulin secretion and sensitivity [65]. Individuals with MetS display a characteristic imbalance of their adipokine profile, which leads to changes in insulin sensitivity and biochemical alterations of metabolites, making an individual more prone to metabolic disorders [66]. In MetS, abdominal obesity appears to precede the appearance of the other MetS components (dyslipidemia, hypertension, and hyperglycemia) [67], where insulin resistance, chronic inflammation, and ectopic fat accumulation are followed by adipose tissue saturation [68]. The expansion of adipose tissue leads to an inflammatory response in the fat tissue due to the infiltration of macrophages and other immune cells, which release proinflammatory cytokines (i.e., tumor necrosis factor-alpha (TNF-α) and interleukin 6 (IL-6)) [69,70].

Previously, a direct relationship between the duration of lactation and both ghrelin and protein-peptide YY (PYY) was revealed 3 years postpartum [54]. Both ghrelin and PYY influence metabolism and appetite regulation. Ghrelin plays a key role in appetite, energy homeostasis, and glucose regulation and has been reported to exert anti-inflammatory effects on macrophages [71,72]. Still, such effects may be hindered in obese, which has been revealed in male mice [72]. High ghrelin levels have been associated with the reduced risk of MetS and DM2 [73]. PYY signals to the brain to attenuate food intake, anxiety, and depression-related behavior and is thought to be a satiety signal [74], reducing food intake [75].

Thyroid function is one of the several factors acting in concert to determine body weight, where even a slightly elevated serum thyroid stimulating hormone (TSH) levels might increase the occurrence of obesity [76]. TSH influences how much T3 (triiodothyronine) and T4 (thyroxine) release into the bloodstream. The positive association between longer duration of lactation and higher levels of thyroid hormone has been revealed among women with a history of GDM, which is a high-risk population for subsequent metabolic complications. In that study, a longer duration of lactation is associated with higher serum fT3 levels and fT3:fT4 ratio, more than 10 (9–16) years after their first pregnancy [77]. T3 level has been found to be a significant predictor of long-term CV mortality, where a higher T3 level is independently associated with a lower risk for sudden death [78]. Conversely, higher fT3 levels and higher fT3:fT4 ratio have been associated with various markers of unfavorable metabolic profile and cardiovascular risk [79].

Pregnancy and lactation may have long-term consequences on energy homeostasis in mothers. In an animal study [80], reproductively experienced mice maintained a higher body weight after weaning compared with age-matched control mice, even though there was no difference in daily food intake or the feeding response to exogenous leptin administration. However, the reproductively experienced mice were less active than age-matched control mice. Interestingly, only the reproductively experienced mice had impaired glucose tolerance after consuming a high-fat diet, which might be due to increased susceptibility to the negative consequences of a high-fat diet after pregnancy and lactation [80]. In humans, both higher energy intake and a higher level of physical activity have been revealed in women with longer duration of breastfeeding (≥10 months), compared to those who lactate for less than 10 months (average duration of lactation per child) [61]. Still, women who lactate for less than 10 months have significantly lower education levels (14 vs. 16 years, *p* = 0.007), which may have inflicted the results, as those with less education may have an unhealthier health behavior. Interestingly women, who lactate for less than 10 months, have a significantly lower intake of protein (69.4 vs. 78.2 g/d) and a lower intake of fiber (13.9 vs. 17.1 g/d), compared to women who lactate for 10 months or more [61].

During pregnancy, the pancreatic islets grow to match dynamic physiological demands, possibly due to prolactin, a hormonal regulator of pregnancy, which represses islet menin levels and stimulated b-cell proliferation [81]. Lactating women have been found to have lower circulating glucose as well as lower insulin secretion, despite increased glucose production rates [57]. Lower glucose levels have also been revealed by others, especially in women with longer duration of lactation (≥10 months), compared to those with an average duration of lactation <10 months (5.0 vs. 5.2 mmol/L, *p* = 0.04), even several years after weaning [61]. The study has also revealed lower serum triglyceride (0.66 mmol/L vs. 0.91 mmol/L, *p* = 0.001) and serum cholesterol (4.32 mmol/L vs. 4.78 mmol/L, p = 0.004), in addition to lower waist-to-hip ratio (0.77 vs. 0.81, *p* = 0.001), in women with a longer duration of lactation [61].

### 4.3. Risk Assessment and Follow-Up Post-Pregnancy

It is suggested that cardiac risk factor identification and management should begin at age 20 years, and lifestyle measures to reduce risk should be repeated at every patient encounter. For women, pregnancy provides an opportune time to evaluate risk [82]. Cardiac risk factors (e.g., hypertension, smoking, obesity, diabetes, pre-eclampsia) are routinely assessed during antenatal care, and midwives and obstetricians are often the first health care personnel to recognize women with elevated cardiac risk profiles [83]. Despite midwifery care is associated with reduced maternal mortality and morbidity and improved public health outcome [84], midwives and obstetricians have rarely been considered a part of the health care service, which focuses on women‘s health beyond the postpartum period. There is a potential role for midwives and the obstetric team to collaborate with multidisciplinary teams to facilitate cardiac risk assessment to ensure optimal management to reverse post-pregnancy perturbations [82,85]. MetS combined with adverse pregnancy outcomes, such as preeclampsia, is associated with a 4 to 8 times increase in CVD-related death [82], emphasizing the need for structured programs like maternal health clinics for post-partum cardiovascular risk screening and tailored follow-up care [85].

Our results indicate that promotion and sustaining of breastfeeding should be prioritized in follow-up care for women post-pregnancy. Despite a recent discourse paper challenges the promotion and breastfeeding and contests the long-term health benefits [86], our results are in line with emerging evidence, supporting lactation as beneficial for women.

### 4.4. Limitations

The systematic literature review is pivotal for advanced understanding and evidence-based decision-making [87]. Our focused review of the evidence is based on conventional systematic review strategies: sensitive searching, systematic screening, and independent quality assessment. Regarding the findings in our review, we cannot draw any definite conclusion about the outcome of the reviewed studies. The majority of studies were cross-sectional, and the eligibility criteria in the reviewed studies were kept broad to include a sample that is similar to the type of people under management in the general population [88]. However, this also generates more uncertainty; it is not known to what extent the women included were representative of the background population, ethnicity was seldom addressed, and in half of the studies, participants were unclearly described. Confounding factors were unclearly assessed in three studies. Confounding occurs when an independent variable is associated both with the variables of interest and the outcome (e.g., age) [88], and hidden confounders (e.g., comorbidities, parity) may have affected the results. The findings should also be interpreted with caution, given the variation between studies. There was evidence of substantial between-study-heterogeneity; however, we were not able to test for the funnel plot asymmetry as it is not recommended when there are fewer than 10 studies in the meta-analysis [89]. Consequently, reporting bias cannot be ruled out because the test power is too low to distinguish change from real asymmetry. As a funnel plot asymmetry test was not appropriate, we were not able to examine whether a correlation between study size and MetS outcomes occurred. However, the random effects estimate was not more beneficial than the fixed effects estimates, indicating that breastfeeding was not proven to be more beneficial in smaller studies. Moreover, methodological quality assessment measures did not differ substantially between smaller and larger studies, indicating that it was appropriate to include both smaller and larger studies in the meta-analysis [89].

Randomized controlled trial (RCT) is the most rigorous method to establish the benefits of health behaviors [90]. However, to establish the benefits of breastfeeding from RCTs raises ethical concerns when patients in the control arm of such a trial would receive a suboptimal treatment, and this may explain why we were not able to find and include any RCTs in the current study. Often observational studies recruit a less selected study population than randomized controlled trials (e.g., individuals with comorbidities). Our review included a limited number of studies, which might pose a high risk of bias and have the potential to overestimate the post-pregnancy incidence of MetS and the benefit of lactation, particularly since we were unable to include all studies in the quantitative analyses, as three studies lacked sufficient information about MetS outcomes. However, the findings related to MetS and lactation are supported by previous reviews.

## 5. Conclusions

The results from this review and meta-analysis suggest that lactation may have a preventive role in MetS; however, the pooled effect was non-conclusive. Future studies are needed to elucidate the role of breastfeeding on MetS.

## Figures and Tables

**Table 1:** Inclusion and exclusion criteria.

PICOS	Inclusion Criteria	Exclusion Criteria
Population	Adult women	Animal studies
Intervention	Breastfeeding	Lab studies Not breastfeeding
Comparison	Duration of breastfeeding	No duration of breastfeeding
Outcome	MetS (prevalence or incidence)	No established definition of MetS
Study design	Cross-sectional, prospective cohort, and intervention studies	Abstracts and protocols

MetS: Metabolic syndrome. PICOS: Population, intervention, comparison, outcome, study design.

**Table 2:** Prospective studies on breastfeeding and metabolic syndrome (MetS).

Study	Participants	Adjustments	Results
Gunderson et al., 2010 [38], 20-year follow-up, CARDIA (1986–2006)	*n* = 1399 18–30 years, nulliparous and free of MetS at baseline, NCEP ATP III, 0–1 month, >1–5 months, 6–9 months, and >9 months, cumulative lactation duration for all births within intervals	Unadjusted Fully adjusted (study center, race, age, education, smoking, parity, BMI, MetS components, physical activity)	Longer duration of lactation inversely associated with the incidence of MetS (>1–5 months, 6–9 months, >9 months) compared with <1 month (relative hazard range non-GDM 0.40–0.49 and GDM groups 0.11–0.28) all *p* < 0.001 The stronger association among GDM in a fully adjusted model (relative hazard range 0.14–0.33) than non-GDM (relative hazard range 0.44–0.61), all *p* = 0.03
Ramezani Tehrani et al., 2014 [39], 9-year follow-up, TLGS	*n* = 925 15–50 years without MetS at baseline, the lifetime duration of lactation: none, 1–6, 7–12, 13–23, or ≥24 months	Age, physical activity, daily caloric intake, BMI, and parity	13–23 months lifetime duration of lactation associated with a higher incidence of MetS compared with 24 months or more RR 1.8 (95% CI 1.0–3.4) (adjusted)

CARDIA: Coronary Artery Risk Development in Young Adults. TLGS: Tehran Lipid and Glucose Study. GDM: Gestational diabetes mellitus. NCEP ATP III: National Cholesterol Education Program Adult Treatment Panel III. BMI: Body Mass Index.

**Table 3:** Cross-sectional studies on breastfeeding and metabolic syndrome (MetS).

Reference	Population	Adjustments	Results
**Cho et al., 2009** [44]	***n* = 892** **postmenopausal women KNHANES (2005)**	**Age, BMI, demographic, socioeconomic, lifestyle factors**	**No association**
Choi et al., 2017 [40]	*n* = 4724 aged 19–50 years KNHANES (2010–2013)	Age, BMI, household income, educational level, marriage status, smoking, alcohol, physical activity, age at menarche, menopause, parity, oral contraceptives	Lactation ≥12 months inversely associated with MetS 6–11 months OR 0.91 (95% CI 0.67–1.24) 12–23 months OR 0.73 (95% CI 0.56–0.95) ≥24 months OR 0.70 (95% CI 0.53–0.92) compared with ≤5 months (adjusted)
Cohen et al., 2006 [22]	*n* = 4699 NHANES III	Age, ethnicity, education, income, parity, employment, physical inactivity, alcohol use, smoking, oral contraceptives, postmenopausal hormone therapy	Lactation ≥1 month inversely associated with MetS OR 0.78 (95% CI 0.61–0.99) (adjusted)
Ki et al., 2017 [41]	*n* = 6621 postmenopausal women KNHANES 2010–2012		Lower prevalence of MetS in women who breastfed ≥6 months compared to those who had not breastfed (29.5 (2.1) vs. 88.4 (4.6), *p* = 0.056)
**Kim et al., 2016** [45]	***n* = 1053** **parous women 30–49 years** **KNHANES V-1 (2010)**	**Income, education level, exercise, the last childbirth age**	**No association** **Women in their 30s OR 0.99** (**95% CI 0.95–1.02); Women in their 40s OR 0.98** (**95% CI 0.94–1.02)** **(all adjusted)**
**Moradi et al., 2016** [46]	***n* = 978** **reporting live birth**	**Age, age at first pregnancy, number of pregnancies, DM, hypertension**	**No association** **OR 0.99 (95% CI 0.99–1.00) (adjusted)**
Ram et al., 2008 [42]	*n* = 2516 reporting live birth	Age, smoking, parity, ethnicity, socioeconomic status, study site, physical activity, caloric intake, high-school BMI	Duration of lactation inversely associated with MetS OR per each additional year of lactation 0.88 (95% CI: 0.77, 0.99) Ever having breastfeed associated with decreased risk of MetS, adjusted OR 0.77 (95% CI 0.62, 0.96) compared to not having breastfeed (all adjusted)
Yu et al., 2019 [43]	*n* = 622 Parous women ≥ child with pregnancy complications, 6–12 months postpartum last-child	Age, ethnicity, education, income, smoking, parity, physical activity, time postpartum, BMI, gestational weight gain	Increased breastfeeding duration decreased the likelihood of MetS (adjusted) OR 0.89 (95% CI 0.79–0.99)

The studies that revealed no association between breastfeeding and MetS in women are written in bold. CARDIA: Coronary Artery Risk Development in Young Adults. KNHANES: The Korea National Health and Nutrition Examination Survey. NHANES III: Third National Health and Nutrition Examination Survey. BMI: Body mass index, DM: Diabetes mellitus.

**Table 4:** Different criteria for the duration of lactation used in studies included in this review.

Reference	Criterion
Gunderson et al., 2010 [38]	Total (i.e., 0–1 month, >1–5 months, 6–9 months, and >9 months) Nulliparous 18–30 years and free of MetS at baseline (20y follow-up)
Ramezani Tehrani et al., 2014 [39]	Total (none, 1–6 months, 7–12 months, 13–23 months, and ≥24 months) 15–50 years at baseline. Among the 340 non-lactating women, 311 were nulliparous
**Cho et al., 2009 [44]**	**Total < or ≥ 1 month**
Choi et al., 2017 [40]	Total (≤5, 6–11, 12–23, or ≥24 months)
Cohen et al., 2006 [22]	Total < or ≥ 1 month
Ki et al., 2017 [41]	Total per child (<1, 1–6, 7–12, 13–18, and >18 months)
**Kim et al., 2016 [45]**	**Breastfeeding experience: yes/no**
**Moradi et al., 2016 [46]**	**Lifetime lactation duration after all deliveries (month)**
Ram et al., 2008 [42]	Number of months per child
Yu et al., 2019 [43]	Last child (none, < 6 or ≥ 6 months)

The studies that revealed no association between breastfeeding and MetS in women are written in bold. Total: Total lifetime duration of lactation.

**Table 5:** Different criteria for waist circumference that were used to diagnose metabolic syndrome (MetS) in the included studies in this review.

	Criteria	Study	Sample Population
Waist	>80 cm	Choi et al., 2017 [40]	Korean
		Ki et al., 2017 [41]	Korean Asian women
		Ram et al., 2008 [42]	Chinese/Japanese
	≥85 cm	**Cho et al., 2009 [44]**	**Korean**
		**Kim et al., 2016 [45]**	**Korean**
	>88 cm	Cohen et al., 2006 [22]	US, multi-ethnic
		Gunderson et al., 2010 [38]	US, multi-ethnic
		**Moradi et al., 2016 [46]**	**Iran**
		Ram et al., 2008 [42]	US/Canada, multi-ethnic
		Yu et al., 2019 [43]	Canada
	≥95 cm	Ramezani Tehrani et al., 2014 [39]	Iran

The studies that revealed no association between breastfeeding and MetS in women are written in bold.

## Supplementary Materials

The following are available online at https://www.mdpi.com/2072-6643/12/9/2718/s1, File S1: The PRISMA statement. S2: Quality Assessment.
