# Supplementary material for: Duration of Lactation and Maternal Risk of Metabolic Syndrome: A Systematic Review and Meta-Analysis
Source: Nutrients. 2020 Sep 5;12(9):2718. doi: 10.3390/nu12092718 (PMC7551509; doi:10.3390/nu12092718)
Supplement: Supplementary file 1 [file nutrients-12-02718-s001.zip › Supplemental/Supplemental S2 Quality Assessment.pdf]

The Joanna Briggs' Checklist for Cross-Sectional Studies

Author, year	Were the criteria for inclusion in the sample clearly defined?	Were the study subjects and the setting described in detail?	Was the exposure measured in a valid and reliable way?	Were objective, standard criteria used for measurement of the condition?	Were confunding factors identified?	Were strategies to deal with confunding factors stated?	Were the outcomes measured in a valid and reliable way?	Was appropriate statistical analysis used?
Yu, 2019	Yes	Yes	Yes	Yes	Yes	Yes	Yes	Yes
Ki, 2017	Unclear	Unclear	Yes	Yes	Yes	Yes	Unclear	Yes
Cho, 2009	Yes	Unclear	Yes	Yes	Unclear	Unclear	Yes	Yes
Ram, 2008	Yes	Yes	Yes	Yes	Unclear	Unclear	Yes	Yes
Cohen, 2006	Unclear	Yes	Yes	Yes	Yes	Yes	Yes	Yes
Moradi, 2016	Unclear	Unclear	Yes	Yes	Unclear	Unclear	Yes	Yes
Kim, 2016	Unclear	Unclear	Yes	Yes	Yes	Yes	Yes	Yes
Choi, 2017	Yes	Unclear	Yes	YEs	Yes	Yes	Yes	Yes

The Joanna Briggs' Checklist for Cohort Studies

Author, year	Were the groups similar and recruited from the same population?	Were the exposures measured similarly to assign people to both exposed and unexposed groups?	Was the exposure measured in a valid and reliabe way?	Were confunding factors identified?	Were strategies to deal whit confunding factors stated?	Were the groups/participants free of the outcome at the start of the study?	Were the ouctomes measured in a valid and reliable way?	Was the follo-up time reported and sufficient to be long enough for outcome to occur?	Was the follow-up complete, and if not, were the reasons to loss to follow-up described and explored?	Were strategies to address incomplete follow-up utilized?	Was appropriate statistical analysis used?
Gundersen, 2010	Yes	Yes	Yes	Yes	Yes	Yes	Yes	Yes	Yes	NA	Yes
Thearani, 2014	Unclear	Yes	Yes	Unclear	Unclear	Yes	Yes	Yes	Unclear	Unclear	Yes
